# New Insights in Paediatric Dermatopathology—2nd Edition

**DOI:** 10.3390/dermatopathology11040040

**Published:** 2024-12-17

**Authors:** Sylvie Fraitag

**Affiliations:** Paediatric Dermatopathology Unit, Department of Pathology, Hôpital Necker-Enfants Malades, APHP, 75015 Paris, France; sylvie.fraitag@aphp.fr

Paediatric dermatology is still an expanding subspeciality, which is well illustrated by the growing number of books and articles that have been published on this subject in recent years [1,2,3,4,5,6,7,8,9,10,11,12,13,14]. In 2021 we published a Special Issue on this topic, which met with great success, dealing with ichthyoses [4], blisters in infancy [5], autoinflammatory diseases in children [6], skin lesions in tuberous sclerosis complex [7], superficial spindle cell mesenchymal tumours in children [8], cutaneous melanomas arising during childhood [9], panniculitis in children [10], congenital cystic masses of the neck [11], pseudomalignancies in children [12], multiple papulonodular skin lesions on newborns [13] and venous malformations in childhood [14]. All these articles were based on anatomo-clinical correlation and updates. They also aimed to advise on how to deal with biopsies on young children, in particular neonates in different situations, and to provide examples of typical patterns and clues for a wide range of disorders and how to avoid pitfalls in their treatment.

This second Special Issue will once again cover a wide panorama of paediatric dermatological conditions not previously treated in the first one. In the same way, it will also address dermatologists and pathologists/dermatopathologists interested in paediatric dermatopathology. The articles will be written by famous and experienced pathologists/dermatopathologists together with clinicians.

The complex and difficult subject of cutaneous vasculitis in children will be covered by S Leclerc-Mercier and A Welfringer. They will highlight certain updates, such as cutaneous vasculitis in autoinflammatory diseases [15]. An update on cutaneous lymphoma in children will be given by P Drabent and A Welfringer, meeting the latest Classification of Hematolymphoid Tumours of the WHO 2024, in which some new entities are described [16]. Melanonychia, although rare in children [17,18], is always a challenging and stressful problem and requires not only an experienced pathologist in this subject but also a technical team accustomed to handling small nail samples. It will be treated by I Moulonguet, who is highly experienced in the topic. The category of disorders of mucous membranes in children covers a wide range of diseases spanning from oral cavity lesions to genital ones—subjects that are very rarely treated in the literature from a histopathological point of view. F Plantier, who is highly specialised in these disorders, has chosen to treat, among the existing entities, those which are of real interest to the pathologist: bullous diseases, genetically transmitted diseases, infectious diseases (condyloma acumimatum, Lipschütz genital ulcer [19,20]) and chronic inflammatory diseases, with an emphasis on the signs that might make us think of systemic/auto-inflammatory diseases (Behçet’s disease or gastro-intestinal disorders, such as Crohn’s disease) [21,22]. In addition, nodular lesions in the oral cavity and the vulva and oral and genital pigmented lesions, will be addressed.

Cutaneous mucinoses in children are a heterogeneous group of diseases characterised by the abnormal deposition of mucin in the skin. This is the main histopathological clue resulting in distinctive clinical presentations. They are uncommon in children, in whom diagnoses are difficult. This topic will be covered by the best specialist in this field, F Rongioletti, and by myself [23].

In 2010, a new classification of inherited ichthyoses was released that is still used throughout the world [24]. Among them, keratinopathic ichthyoses are interesting for dermatopathologists to know about as they are easily recognisable through the microscope and can even be classified by morpholological analysis. This is an interesting point to underline at a time when genetic study is becoming a major tool in the context of genodermatoses. Microscopic analysis is easy, rapid and cheap, and S Leclerc-Mercier, one of the best specialists in this field, will demonstrate the usefulness of this method in the field of keratinopathic ichthyoses. In the same way, D Metze will cover the spectrum of desmosomal genodermatoses and will point out the role of the microscopic analysis of some palmoplantar keratodermas; indeed, these can be a warning sign of severe arrhythmogenic cardiopathy [25,26]. Finally, we will address the challenging problem of proliferative nodules in congenital melanocytic naevus. The dermatopathologist’s role is still crucial in distinguishing a proliferative naevus from a malignant melanoma arising in a congenital melanocytic naevus [27]. However, there are now ancillary techniques which can help in such diagnoses, and we will discuss their respective roles.

Hopefully this second edition will be as successful as the first.
